# Tracing genetic resurrection of pointing dog breeds: Cesky Fousek as both survivor and rescuer

**DOI:** 10.1371/journal.pone.0221418

**Published:** 2019-08-26

**Authors:** Silvie Neradilová, Laurie Connell, Pavel Hulva, Barbora Černá Bolfíková

**Affiliations:** 1 Department of Animal Science and Food Processing, Faculty of Tropical AgriSciences, Czech University of Life Sciences, Prague, Czech Republic; 2 Molecular & Biomedical Sciences, University of Maine, Orono, Maine, United States of America; 3 Department of Zoology, Faculty of Science, Charles University, Prague, Czech Republic; 4 Department of Biology and Ecology, University of Ostrava, Ostrava, Czech Republic; University of Iceland, ICELAND

## Abstract

Cesky Fousek is considered to be one of the oldest pointing dog breeds in Europe and has been appreciated for its versatile working skills. Because it faced extinction in the past, the Cesky Fousek was restored from German Wirehaired and Shorthaired Pointers. Additionally, the breed was recently used in the USA with the initial intent of improvement of the Wirehaired Pointing Griffon (synonymous with Korthals Griffon) by the Bohemian Wirehaired Pointing Griffon Club of America. This study evaluates genetic diversity parameters of Cesky Fousek and compares them to the other continental pointing dogs that played a role in the formation of its gene pool. DNA from buccal swab and blood samples (*n* = 405) were analyzed using 18 microsatellite markers. Parameters of genetic polymorphism show that the Cesky Fousek breed has a comparable rate of variation as other hunting breeds despite the low population size and severe historical bottlenecks. Clustering analyses reveal a unique genetic status as a distinct pointing dog breed and the relatedness of the breeds is in good concordance with historical data. The present study demonstrates that despite historical admixture among lineages, separate pointing breeds constitute genetically differentiated units, mirroring unique breeding stocks and pedigree isolation among specific breed clubs, reflecting differences in breeding programs under each association.

## Introduction

Dogs (*Canis familiaris*) are the first domesticated animals that interacted with humans from the Paleolithic [1,2]. The only ancestor is a grey wolf (*C*. *lupus*) [3–5]. It is likely that humans used the first dogs for protection of resources and for hunting [6]. Nowadays, we recognize more than 400 dog breeds [7], most appearing from the mid-19th century [8]. Their origin is associated, for example, with the rise of the middle class, nationalism, the industrial revolution, and establishment of Mendelian genetics [9]. At the beginning of breed development, the gene flow among many breeds was probably extensive [9,10]. Pedigree isolation in modern breeds was often connected with strong founder effects, genetic drift, and inbreeding, resulting in decreased genetic diversity and pronounced effect of the deleterious recessive alleles [11,12]. Moreover, strict artificial selection for a few traits often leads to phenotypes prone to particular health problems. For example, extreme selection for phenotypic traits led to respiratory defects in short-muzzled breeds, spinal problems in breeds selected for long backs etc. [13–15]. Some breeds, for example Irish Wolfhound and Cesky Fousek, went through a severe bottleneck in the near past (e.g. due to armed conflicts) and have been restored from a few founding animals [10,16]. An overuse of popular sires and unequal use of breeding individuals highly influences genetic composition of most breeds. All these phenomena reduce the genetic variability of the breeds and have a negative impact through increased concentration of heritable diseases, especially within the breeds with a small population.

**Fig 1:**
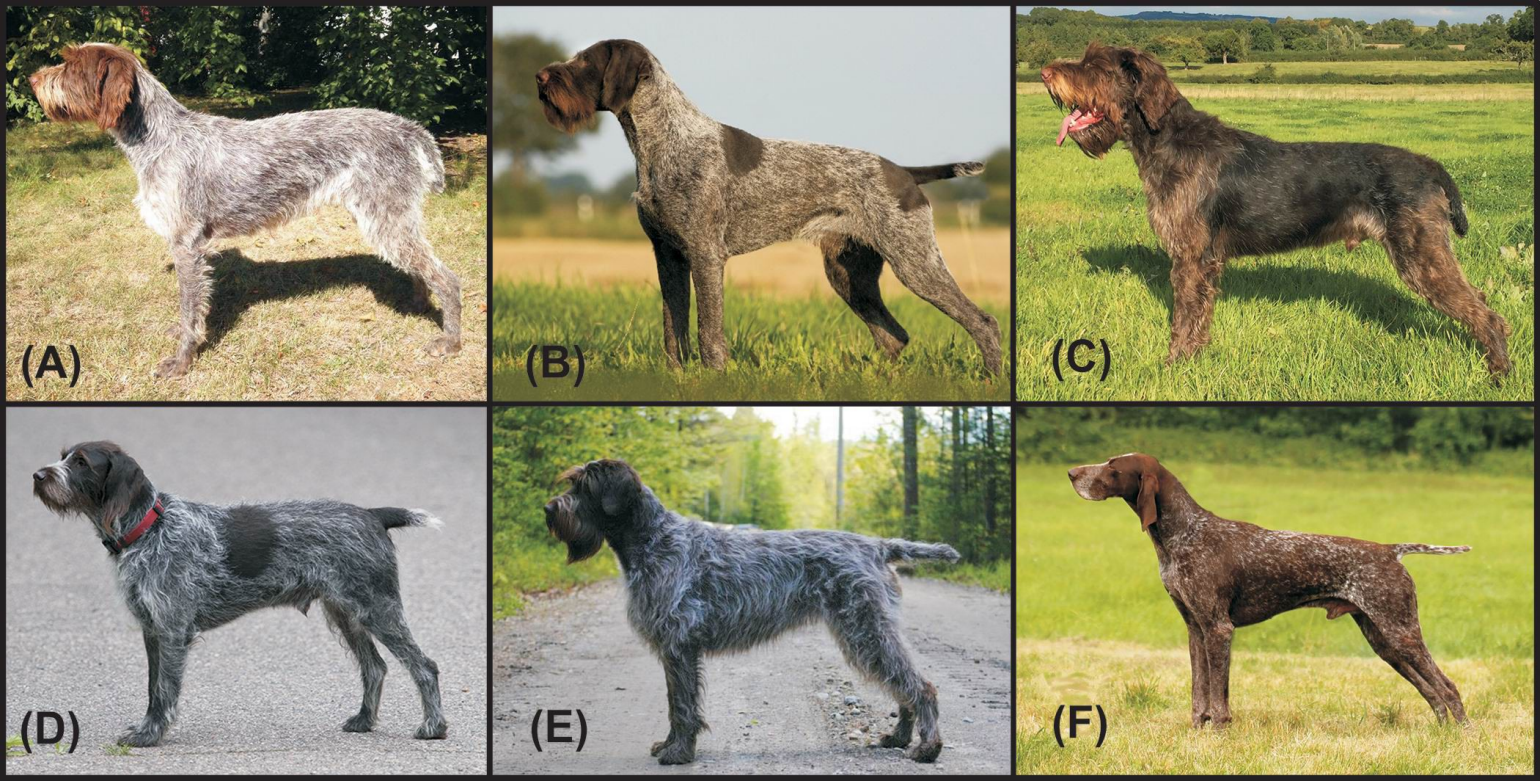
General appearance of six studied breeds. (A) Cesky Fousek; (B) Deutsch Drahthaar; (C) German Wirehaired Pointer; (D) individual of Bohemian Wirehaired Pointing Griffon Club of America; (E) Wirehaired Pointing Griffon; (F) German Shorthaired Pointer. Tail docking is allowed for hunting breeds in the countries of origin for these animals which were: Czech Republic (A; F), Germany (B; C) and USA (D; E).

The present study focuses on pointing dog breeds, representing a substantial part of gun dog variation. We used 18 microsatellite markers to compare the genetic structure and diversity of selected central European pointing breeds (Cesky Fousek–CF; Deutsch Drahthaar–DD; German Wirehaired Pointer–GWP; individuals of Bohemian Wirehaired Pointing Griffon Club of America–BWPGCA; Wirehaired Pointing Griffon–WPG; and German Shorthaired Pointer–GSP; Fig 1). European wirehaired breeds of pointing dogs have a very complex history. Prior to establishment of modern European pointing dog breeds, wirehaired dogs in general were popular among hunters. Their coat was an advantage in cold weather and in dense bush in the woods. However, at that time the breeding was not controlled, the hunters were just selecting dogs with good working skills and the wirehaired individuals were mated together to obtain the best dogs for the hunters [9,17]. At the end of 19^th^ and the beginning of 20^th^ century, when the breeds were already established, the wirehaired breeds were, more or less, still interbred [10,18].

The genetic structure of the modern wirehaired breeds was affected by the dramatic political turnovers of 20th century that changed the country borders several times. For example, litters of CFs born in different parts of contemporary Czech Republic were exported to contemporary Germany and Austria and registered in local stud books as different breeds, such as Deutsch Drahthaar (DD) and Deutsch Stichelhaar (DS) [17]. The World Wars pushed the CF breed to the edge of extinction in the Czech regions. However many descendants of the original Czech dogs still existed in Germany under the breed names of DS and DD. It was decided by the Czech CF breeder club to restore the CF breed using descendants of these DS and DD individuals, thus maintaining the genepool of the old CF breed [10].

Although the English translation of Deutsch Drahthaar is German Wirehaired Pointer, these two breeds are no longer the same. They each have their own breed clubs and registrations and their phenotypes can be different. For example, DD may have a black coat color while it is forbidden in GWP and the GWP may carry white coat color that is forbidden for DD (S1 Fig). Historically, GWPs are from the same background as DDs, but are registered by the American Kennel Club (AKC) and Canadian Kennel Club (CKC). The North American GWPs are now selected primarily for upland and waterfowl hunting (feathered game). The original DDs are recognized by the Federation Cynologique Internationale (FCI) and are usually used for a versatile work with various types of small game in the field, water, and woods. Until recently, DDs are imported to the USA and registered there as GWPs, however, most DD imported now into the USA are registered with the FCI through the German DD club.

There was a great debate in Germany during the late 1880s about discrimination and nomenclature of breeds. At that time the German shorthaired, longhaired, and wirehaired pointers were considered one breed with several variations in coat [19]. By 1888 the GSP and WPG had been formally recognized as breeds and the studbooks closed. The wirehaired breeds in central and northern Europe continued to intermix with occasional crosses with the GSP, a breed that was much more numerous and had faster fieldwork [9,10].

The breed of Wirehaired Pointing Griffon (synonymous with Korthals Griffon) has a very complex history as well. The WPG was developed from mostly German wirehaired dogs, spaniels, and Pointers of various backgrounds and has a French parent breed club [9,20]. The WPG also suffered from reduction of numbers during both World Wars but the French breed club (Club Français du Griffon d'Arrêt a Poil Dur Korthals) maintained a closed studbook [21] unlike the Czech CF club. At the end of 19^th^ century, the WPG breed was introduced in North America where it was bred with a closed studbook until 1985 when then Wirehaired Pointing Griffon Club of America (WPGCA; later changed to Bohemian Wirehaired Pointing Griffon Club of America–BWPGCA) decided, that the breed needed improvement [20,22]. The reasons given leading to this decision were the loss of hunting ability, increased occurrence of the recessive genetic diseases, and the diminishing coat quality. The breed chosen for the restoration was CF and the cross-breeding started in 1985. The improvement was immediate and BWPGCA switched to breeding CF and continues to import Czech CF individuals into its breeding system [19,20]. Subsequent to the switch there were backcrosses to two WPG males (2013 & 2014) to increase genetic diversity. Since 2014 no WPG have been added back into the BWPGCA gene pool. The BWPGCA has separate registration and breeding rules that, similar to the FCI but unlike the AKC or CKC, include hunting ability and conformation testing for breeding approval. Therefore, in our study BWPGCA individuals are separated from Wirehaired Pointing Griffons from North America.

The aim of this study was: i) to assess genetic diversity and describe genetic parameters of the modern Cesky Fousek breed which has a small population size and needs proper genetic monitoring, and moreover has a complex history containing at least two other contemporary pointing dog breeds in its genetic background; ii) to evaluate the level of genetic divergence between the German pointing dog breeds and Cesky Fousek and compare it to the known history of the breeds, and iii) to assess the level of genetic differentiation between Cesky Fousek, North American Wirehaired Pointing Griffons, and the individuals from Bohemian Wirehaired Pointing Griffon Club of America with a recent mixed CF/WPG background.

## Materials and methods

### Ethics statement

The Expert Commission Ensuring Welfare of Experimental Animals working at the Czech University of Life Sciences Prague has approved this study the least invasive, thus, according to the Czech law, this study does not need an approval of a State Ethical Committee.

### Sampling

Samples were collected from 405 individuals representing six pointing breeds: Cesky Fousek (CF; *n* = 193), Deutsch Drahthaar (DD; *n* = 87), German Wirehaired Pointer (GWP; *n* = 26), individuals of Bohemian Wirehaired Pointing Griffon Club of America (BWPGCA; *n* = 38), Wirehaired Pointing Griffon (WPG; *n* = 20) and German Shorthaired Pointer (GSP; *n* = 41), during years 2012–2016. Samples were taken as buccal swabs (FLOQSwabs®) with agreement of the dog owners during dog shows, hunts, and hunting competitions. The origin of the samples is given in S1 Table. Samples from the Czech Republic and the Netherlands were taken by SN, BČB, MJ, and PH (*n* = 255); samples from other countries were obtained directly from owners (*n* = 150). These owners were instructed how to take the samples correctly to avoid contamination. Several samples were obtained from the Cornell Veterinary Biobank (*n* = 27), which provided 23 samples of BWPGCA individuals and four samples of WPG individuals. Some individuals sent by BWPGCA were imported individuals from the Czech Republic; these samples were classed with the pure CFs from the Czech Republic to avoid biased results. DNA from buccal swabs was extracted using Genomic DNA Mini Kit (Geneaid Biotech Ltd., New Taipei, Taiwan) for tissue and saliva according to the manufacturer's protocol.

### PCR and fragmentation analysis

We have selected nuclear microsatellites as the genetic marker type for this study due to their high polymorphism, neutrality in respect to selection and a good statistical power to detect recent population structure. A commercially available microsatellite genotyping kit (Canine Panel 1.1; ThermoFisher Scientific) was used to amplify 18 microsatellite markers (AHTk211, CXX279, REN169O18, INU055, REN54P11, INRA21, AHT137, REN169D01, AHTh260, AHTk253, INU005, INU030, FH2848, AHT121, FH2054, REN162C04, AHTh171 and REN247M2). Fragmentation analysis was processed on ABI Prism 3100 Avant Genetic Analyser (Applied Biosystems) using polymer POP-4tm separation matrix with DS-33 matrix standard size and Gene Scan TM 500 LIZ (Applied Biosystems) size markers.

### Data and statistical analysis

Length of each allele was scored and binned in GENEIOUS R10 (https://www.geneious.com). FSTAT was used to estimate allelic richness (Ar) based on minimal population size from the smallest group in the study (18 individuals). Ar describes genetic variation while eliminating the effect of the sample size. Estimates of expected heterozygosity (H_E_), observed heterozygosity (H_O_), inbreeding coefficient (F_IS_) and number of private alleles for each population were calculated in software GENEALEX 6.501. Pairwise fixation index values (F_ST_) and Hardy-Weinberg (H-W) test for heterozygote deficiency were calculated in GENEPOP software [23,24]. Exact p-values for H-W test were calculated using a Markov chain algorithm [25] with 1000 dememorization steps for 500 batches and 1000 iterations per batch. Software POPULATIONS [26] was used to compute a matrix of minimum genetic distances according to Nei [27] for all individuals. This matrix was used to construct a phylogenetic neighbour-joining tree of relationships among populations and individuals. The tree was graphically visualized in FIGTREE [28]. Visualization of genetic relationships between individuals was processed in GENETIX software [29] using factorial correspondence analysis (FCA). To assign particular genotypes to respective clusters (K) and to assess substructure within the dataset, Bayesian clustering approach implemented in software STRUCTURE [30] was used. Number of tested K ranged from 1 to 10. For each value of K, five runs were performed with a burn-in period of 300 000 and 1 000 000 MCMC (Markov chain Monte Carlo) repetitions. The best support for number of clusters (K) was combined in StructureSelector [31] using Evanno method of ΔK [32] and MedMed K, MedMean K, MaxMed K and MaxMean K statistics [33] which are more accurate for unequal population sample sizes (S2 Fig).

## Results

Each individual had maximally 20% of missing data (S1 Table). All genotypes can be found in S1 Table. The highest number of alleles per locus (Na) was found in DD (Na = 6.222; Table 1). The highest Ar value was found in GSP (Ar = 5.304) and the lowest in BWPGCA (Ar = 4.723), with CF showing an intermediate value of Ar = 5.245 (Table 1). The H_O_ ranged between H_O_ = 0.669 (in CF; Table 1) and H_O_ = 0.639 (in BWPGCA). The highest value of F_IS_ was found in WPG breed (F_IS_ = 0.061; Table 1). The lowest value of F_IS_ was found in GSP breed (F_IS_ = -0.004; Table 1). In CF, F_IS_ = 0.005. Values of Hardy-Weinberg heterozygote deficiency test show that there is a significant lack of heterozygotes in DD.

**Table 1:** Descriptive genetic parameters for all studied breeds.

Breed	n	Na	Ar	H_E_	H_O_	HWE	Np	F_IS_
**CF**	193	6.111	5.245	0.673	0.669	*	3	0.005
**SE**		0.342		0.026	0.027			0.011
**DD**	87	6.222	5.117	0.676	0.660	***	5	0.022
**SE**		0.308		0.020	0.020			0.012
**GWP**	26	5.278	5.038	0.657	0.652	ns	5	0.014
**SE**		0.321		0.026	0.038			0.038
**BWPGCA**	38	4.889	4.723	0.639	0.639	ns	4	0.002
**SE**		0.241		0.028	0.033			0.028
**WPG**	20	5.222	5.142	0.683	0.644	*	6	0.061
**SE**		0.222		0.029	0.039			0.035
**GSP**	41	5.889	5.304	0.650	0.653	ns	4	-0.004
**SE**		0.322		0.040	0.043			0.022

CF = Cesky Fousek; DD = Deutsch Drahthaar; GWP = German Wirehaired Pointer; BWPGCA = individuals of Bohemian Wirehaired Pointing Griffon Club of America; WPG = Wirehaired Pointing Griffon; GSP = German Shorthaired Pointer; SE = standard error; n = number of individuals; Na = average number of alleles per locus; Ar = allelic richness; H_E_ = expected heterozygosity; H_O_ = observed heterozygosity; HWE = Hardy-Weinberg test for heterozygote deficiency; Np = number of private alleles; F_IS_ = coefficient of inbreeding; The significant values for heterozygote deficiency test are marked with asterisks:

* P<0,05, **P<0,01

*** P<0,001, ns P>0.05.

Values of F_ST_ calculated for each pair of populations are stated in Table 2. The values indicate that the breed of CF is less differentiated from BWPGCA (F_ST_ = 0.030) than from DD and GWP (F_ST_ = 0.086/0.077). The highest differentiation was found between the breed of GSP and BWPGCA (F_ST_ = 0.144).

**Table 2:** Pairwise differentiation index (F_ST_) for all pairs of studied populations.

Breed	CF	BWPGCA	WPG	DD	GWP
**BWPGCA**	0.030				
**WPG**	0.118	0.135			
**DD**	0.086	0.119	0.116		
**GWP**	0.077	0.110	0.124	0.036	
**GSP**	0.114	0.144	0.117	0.091	0.115

CF = Cesky Fousek; DD = Deutsch Drahthaar; GWP = German Wirehaired Pointer; BWPGCA = individuals of Bohemian Wirehaired Pointing Griffon Club of America; WPG = Wirehaired Pointing Griffon; GSP = German Shorthaired Pointer.

**Fig 2:**
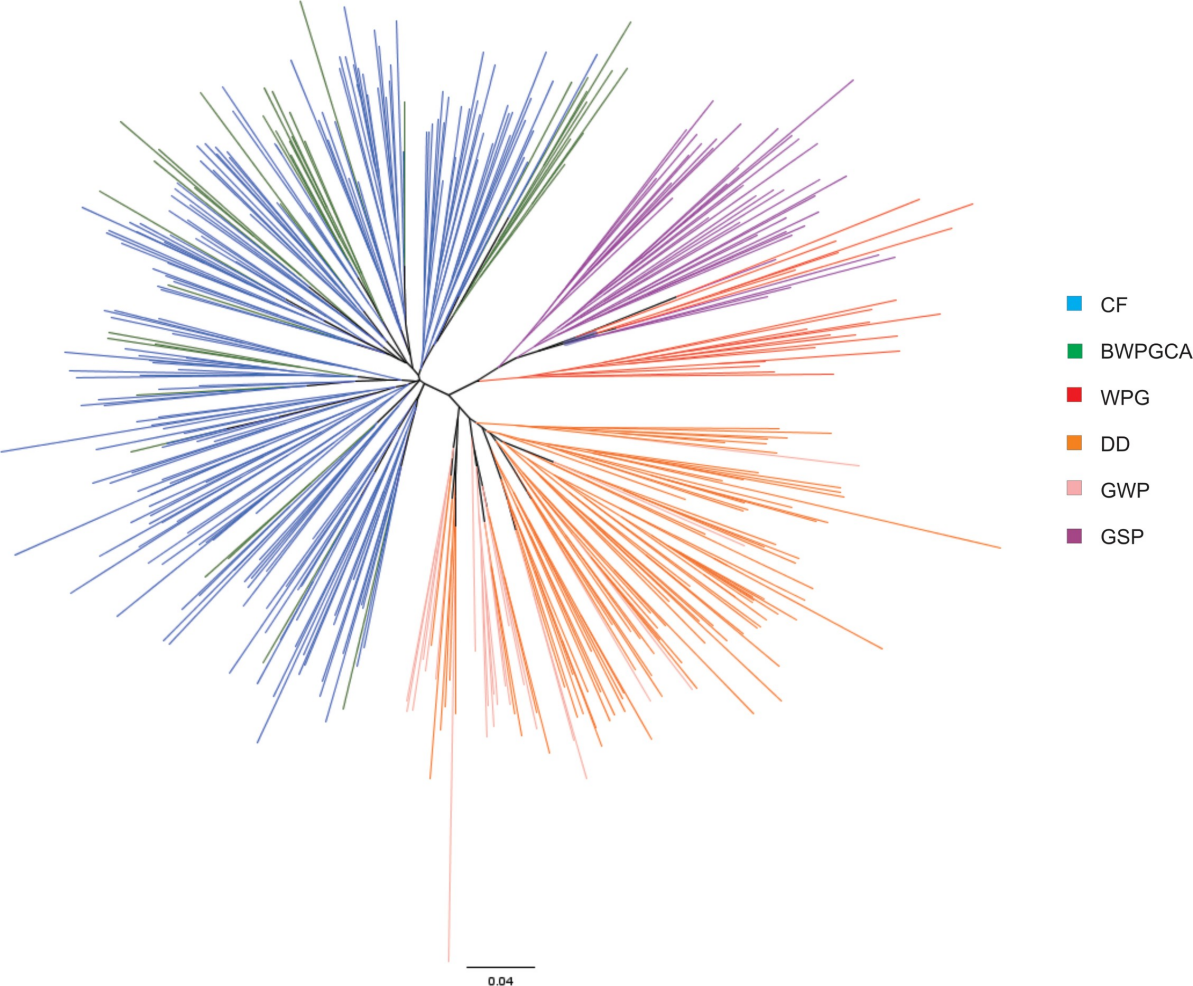
Genealogical tree of individuals based on matrix of minimum genetic distances according to Nei (1972). Blue–Cesky Fousek, dark green–individuals of Bohemian Wirehaired Pointing Griffon Club of America, red–Wirehaired Pointing Griffon, orange–Deutsch Drahthaar, pink–German Wirehaired Pointer, purple–German Shorthaired Pointer.

The genealogical tree shown in Fig 2 proposed three differentiated groups; one containing CF, and BWPGCA, where most BWPGCA are inner lineage of CF. A second group contained WPG and GSP, where some GWP are inner lineage of GSP, but mainly from their own cluster. Last group consisted of DD and GWP (Fig 2).

**Fig 3:**
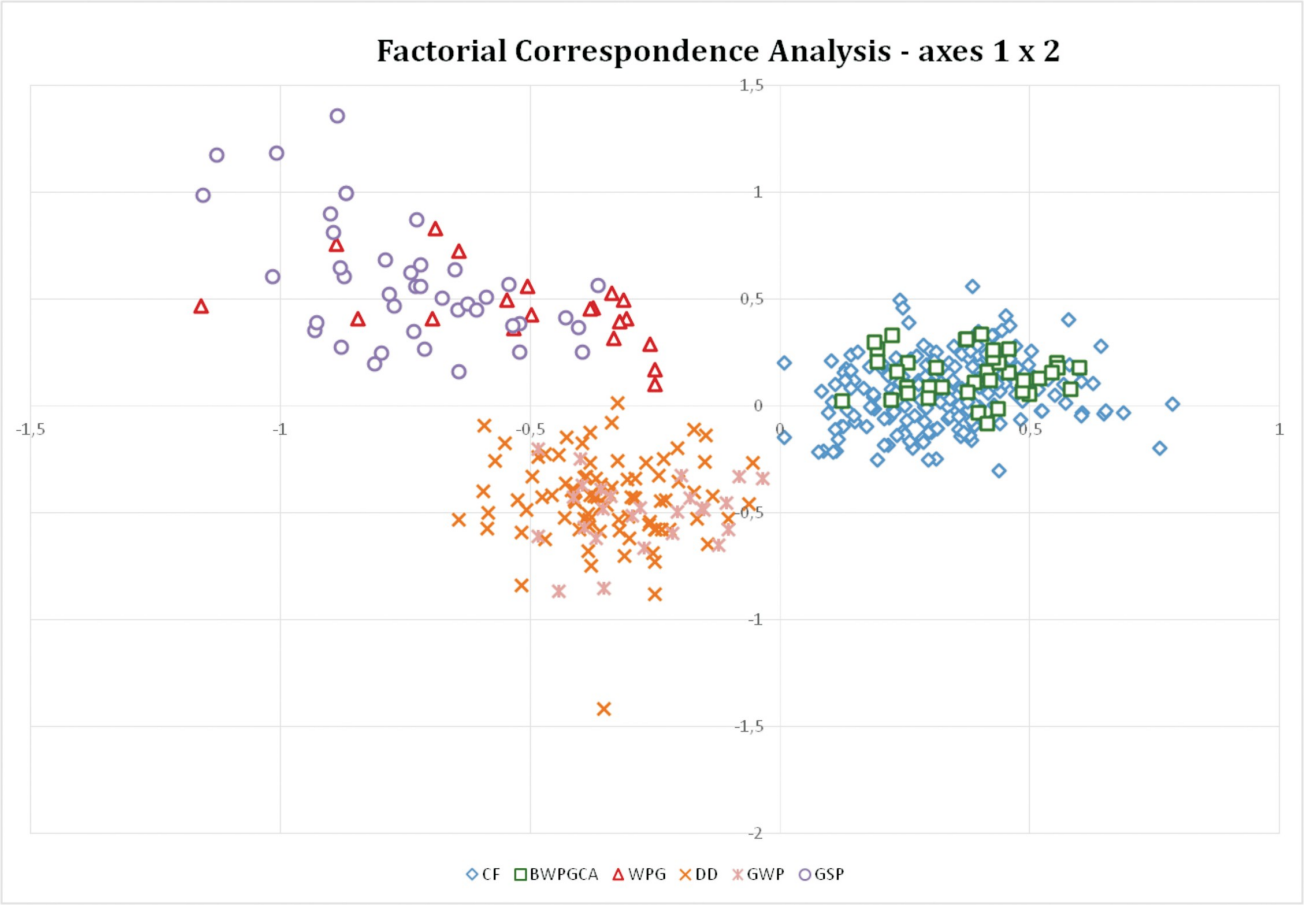
Genetic distances between individuals and populations, based on Factorial Correspondence analysis of 18 microsatellite loci performed in GENETIX software. CF—Cesky Fousek, DD—Deutsch Drahthaar, GWP–German Wirehaired Pointer, BWPGCA—individuals of Bohemian Wirehaired Pointing Griffon Club of America, WPG—Wirehaired Pointing Griffon, GSP—German Shorthaired Pointer.

All three groups are also differentiated by FCA, with particular overlap of clusters (Fig 3). Although it seems that WPG and GSP breeds cluster together, from a different perspective we can see that they are well differentiated (S3 Fig).

**Fig 4:**
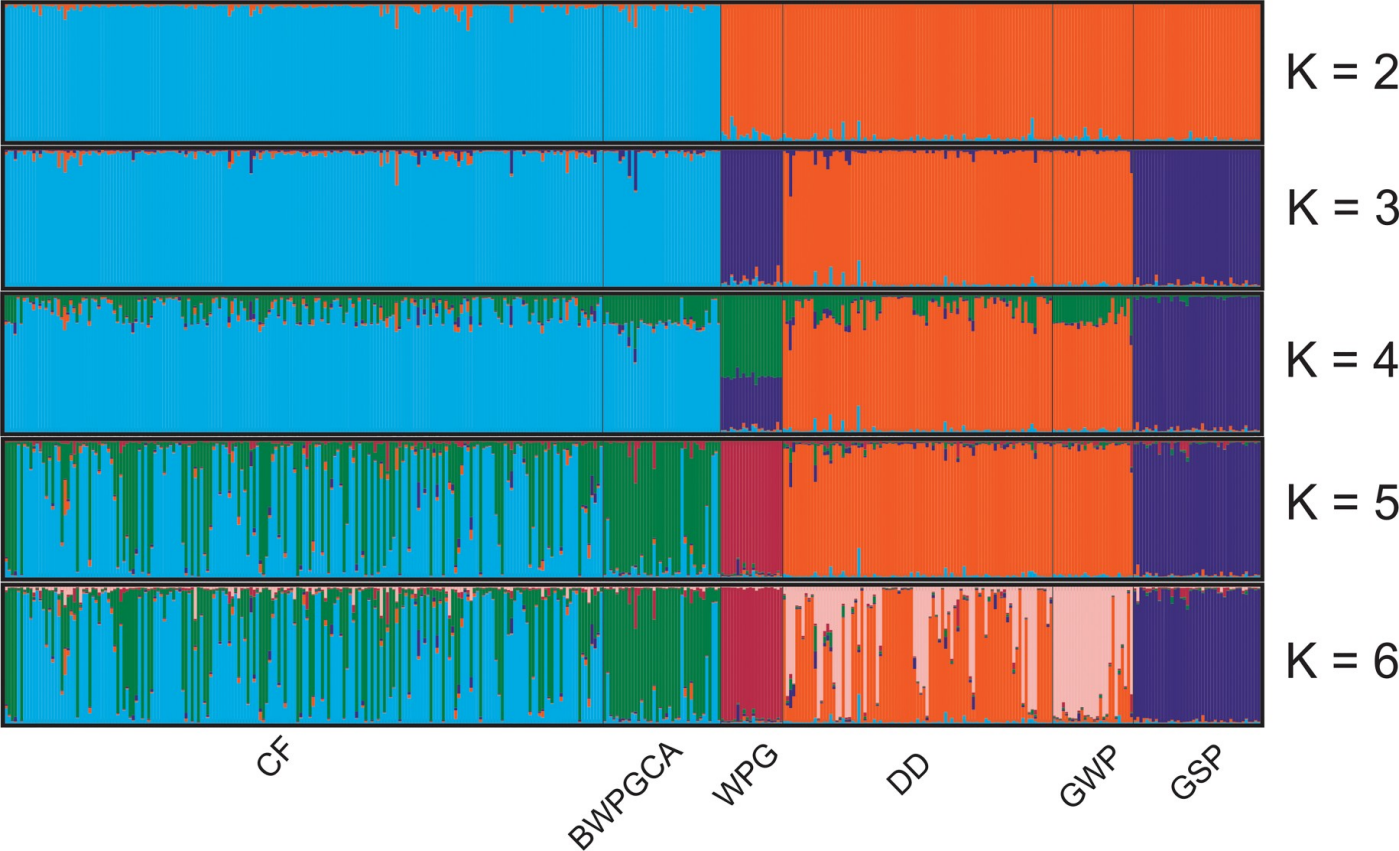
Bayesian clustering analysis of six studied breeds based on 18 microsatellite loci. CF—Cesky Fousek, DD—Deutsch Drahthaar, GWP–German Wirehaired Pointer, BWPGCA—individuals of Bohemian Wirehaired Pointing Griffon Club of America, WPG—Wirehaired Pointing Griffon, GSP—German Shorthaired Pointer.

Higher resolution was achieved using Bayesian clustering analysis in Structure. Using method of Puechmaille [33], the highest support was obtained for *K* = 6 (S2 Fig) where mean membership coefficient for each cluster differentiated all breeds. Considering each individual separately, some degree of shared ancestry between CF and BWPGCA and between DD and GWP is visible (Fig 4). On the other hand, method of Evanno [32] supported *K = 2* as the best number of clusters, where the first group consisted of CF and BWPGCA and the second group consisted of the remainder of the studied breeds (S2 Fig). The first group represented approximately a half of the dataset which might bias the analysis.

## Discussion

Level of genetic diversity evaluated according to the descriptive parameters (such as H_O_, Ar, F_IS_) in this work are similar to those of other studies (S2 Table), although the Cesky Fousek breed exhibits a low level of inbreeding despite the fact that the population is of small size. Moreover, the rate of genetic polymorphism was higher compared to other studied breeds of similar (BWPGCA, GWP, WPG) or larger population size (DD, GSP) (Table 1). Two reasons for this observation could be 1) the recent out-crossing with GSP and DD, and 2) good genetic management of the breed population as a whole. Genetic management of the Czech CF population is based on pedigrees, minimizing the kinship within selected breeding pairs. Breed Wardens select three potential males from which owners of female can select. Such a strong limitations in mate selection are uncommon in other companion animal breeds. Other factors, such as the occurrence of important traits (including hereditary diseases), play a role in the management decisions. Cesky Fousek now has low prevalence of hereditary diseases in contrast to the situation found in many other breeds with a small population size [34].

Since the restoration of the CF, a loose line-breeding method (mating within the breed) is being used and there are currently nine active Czech lines. Each CF line is characterized by different hunting abilities. In order to avoid too close relatedness inside the lines, individuals from different lines are sometimes used to dilute the effect of line-breeding [35,36]. Cross-breeding (mating between different breeds) was used in 2000 when two individuals of different breeds (GSP, DD) were used to improve the hunting abilities of CF. The effect of line-breeding was not clearly visible within present data. We did not test the internal breed structure further because the individual animal membership to a specific line is set according to the pedigrees, can change according to population management needs, and can be different from genetic origin. Some individuals are used in more than one line to increase genetic diversity in that additional line.

Cesky Fousek and DD breeds are phenotypically very similar and for a non-skilled person it is often impossible to discriminate between these breeds. These two breeds were freely mixed together until 1924 when the studbook of DD was closed. In the case of CF, the studbook was closed in 1960. Even though the history of CF is complicated and included genetic rescue from the DD and GSP, our study brings clear evidence that recently the genetic pool of CF is well delimited from these German breeds.

We showed evidence that despite different registration systems for DD and GWP breeds since 1959, both breeds are still close genetically (F_ST_ = 0.036; Figs 3 and 4) although the appearance of the individuals can differ markedly (S1 Fig). High genetic similarity between DD and GWP is related to a high level of admixture between the breeds, as DDs can be imported to North America and registered as GWP under the AKC or CKC. Obviously the selection of different coat colours, which is usually under-laid by a limited number of loci [37], does not outbalance the effects of admixture at the genomic level.

The BWPGCA began crossing CF and WPG in 1985 with increasing CF input since that time. These individuals are more differentiated from the original WPG (F_ST_ = 0.135) than from CF (F_ST_ = 0.030). The position of the BWPGCA animals is not intermediate between CF and WPG but rather shifted toward CF (Fig 3), reflecting different proportions of particular parental breeds within the founding stock. This is a phenomenon described in other mixed breeds such as Czechoslovakian Wolfdogs [38]. Our results evaluated the level of inbreeding in WPG by a value (F_IS_ = 0.061; Table 1) higher than in previous studies [39] where F_IS_ = -0.027. This difference may be the result of different loci used by both studies. It is known that the WPGs display high frequency of recessive genetic diseases (e.g. hip and elbow dysplasia, eye disease, autoimmune thyroiditis) and the natural working abilities were often significantly reduced by a split between show and working sub-populations [20]. The high frequency in disease occurrence and the lack of natural hunting abilities were the original reason of mixing CF with WPG. In this study the dogs managed by BWPGCA have lower inbreeding coefficient than WPG (F_IS_ = 0.002 and F_IS_ = 0.061 respectively; Table 1). Also, the hunting abilities improved significantly compared to the original pre-1985 WPGCA stock [20].

The comparison of results with other studies may be affected due to the different marker sets used (e.g. [39,40]). This issue could be fixed and more information about the history and population structure of these hunting dogs could be gained by using whole-genome data such as genome-wide SNP (single nucleotide polymorphism). Genomic data are especially useful in studying reticulate and adaptive population histories. These data would allow to track the response of the genome to past cross-breeding and following artificial selection in present case. Further increase of sample size and geographic coverage could also deepen the resolution of the demographic reconstructions.

## Conclusions

The history of central European pointing dog breeds is characterized by recurrent founding events and admixture among lineages, for example the Cesky Fousek was used for creation of German wirehaired pointing dogs and later the German dogs were used to restore Cesky Fousek. In turn, Cesky Fouseks were used in the Bohemian Wirehaired Pointing Griffon Club of America to increase genetic variation of the original Wirehaired Pointing Griffon breed. Despite this reticulate demography, gene pools of particular breeds are recently well differentiated, as suggested by clustering analyses. These patterns could be ascribed to unique breeding stocks and pedigree isolation among particular clubs, related to differences in breeding programs under each association.

## Supporting information

S1 Fig Example of pronounced differentiation in coat color in two breeds of the same historical origin—(a) Deutsch Drahthaar; (b) German Wirehaired Pointer.
(PDF)

S2 Fig Output from Structure Selector [32] based on Bayesian clustering in Structure [29]. (TIF)

S3 Fig Studied breeds visualized by Factorial correspondence analysis using different axes (1 x 3) than in Fig 3. (TIF)

S1 Table Origin and genotypes of the samples used for the study. (XLSX)

S2 Table Parameters of genetic variability of hunting breeds gathered from different scientific papers. (PDF)
