# Supplementary material for: Tracing genetic resurrection of pointing dog breeds: Cesky Fousek as both survivor and rescuer
Source: PLoS One. 2019 Aug 26;14(8):e0221418. doi: 10.1371/journal.pone.0221418 (PMC6709920; doi:10.1371/journal.pone.0221418)
Supplement: S1 Fig — Example of pronounced differentiation in coat color in two breeds of the same historical origin—(a) Deutsch Drahthaar; (b) German Wirehaired Pointer. (PDF) [file pone.0221418.s001.pdf]

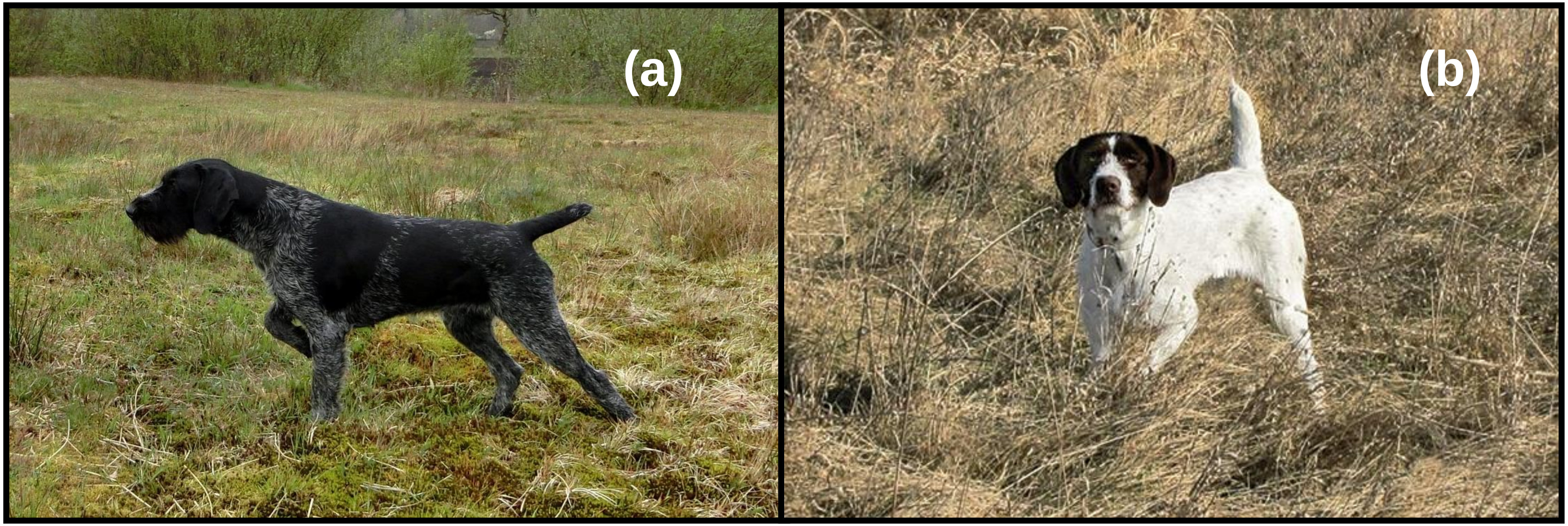

S1 Fig. *Example of high differentiation in coat color in two breeds of the same historical origin - (a) Deutsch Drahthaar; (b) German Wirehaired Pointer.*
