# Supplementary figures and images for: Tracing genetic resurrection of pointing dog breeds: Cesky Fousek as both survivor and rescuer
Source: PLoS One. 2019 Aug 26;14(8):e0221418. doi: 10.1371/journal.pone.0221418 (PMC6709920; doi:10.1371/journal.pone.0221418)

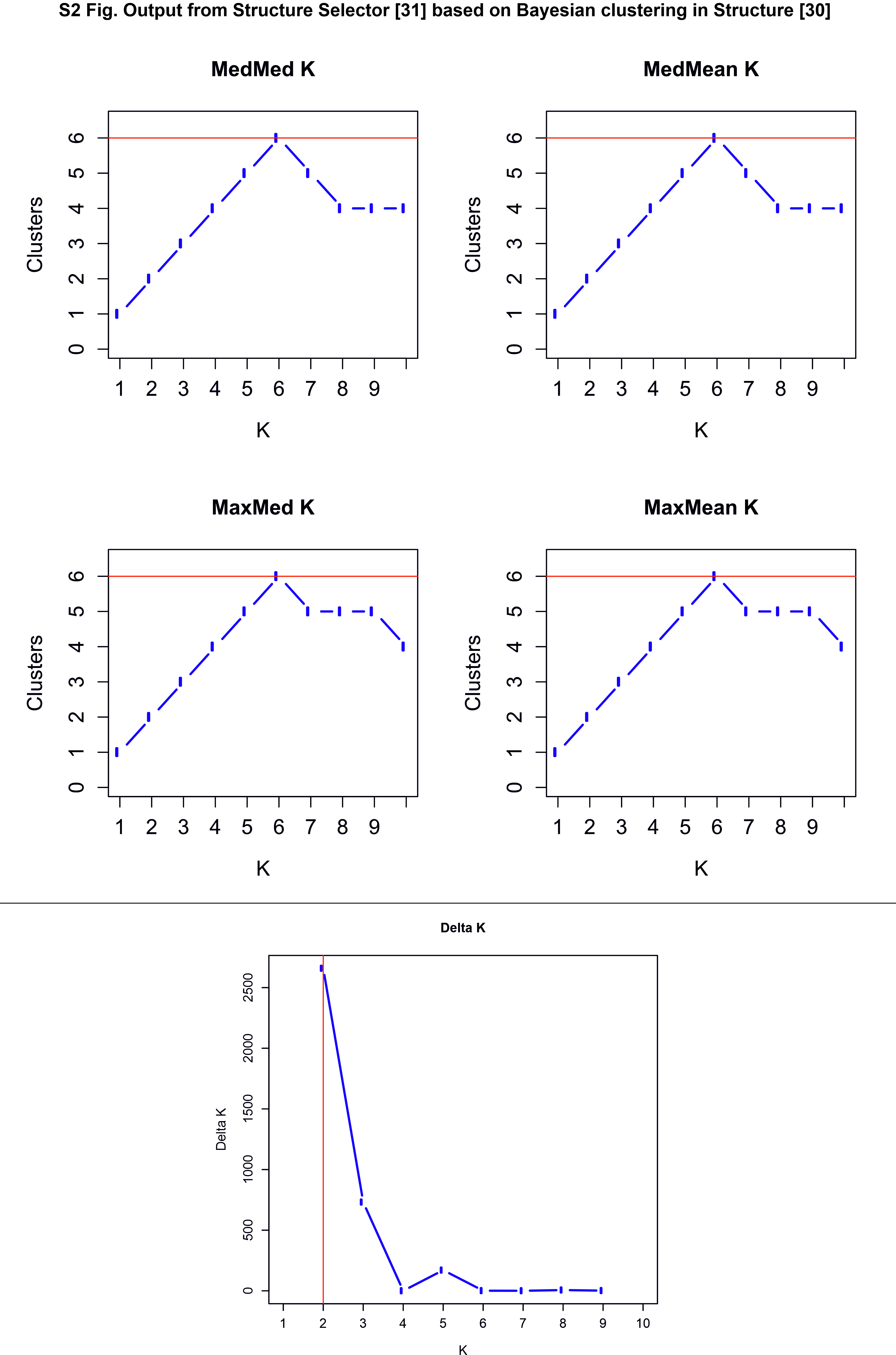

Supplement: S2 Fig — (TIF) [file pone.0221418.s002.tif]

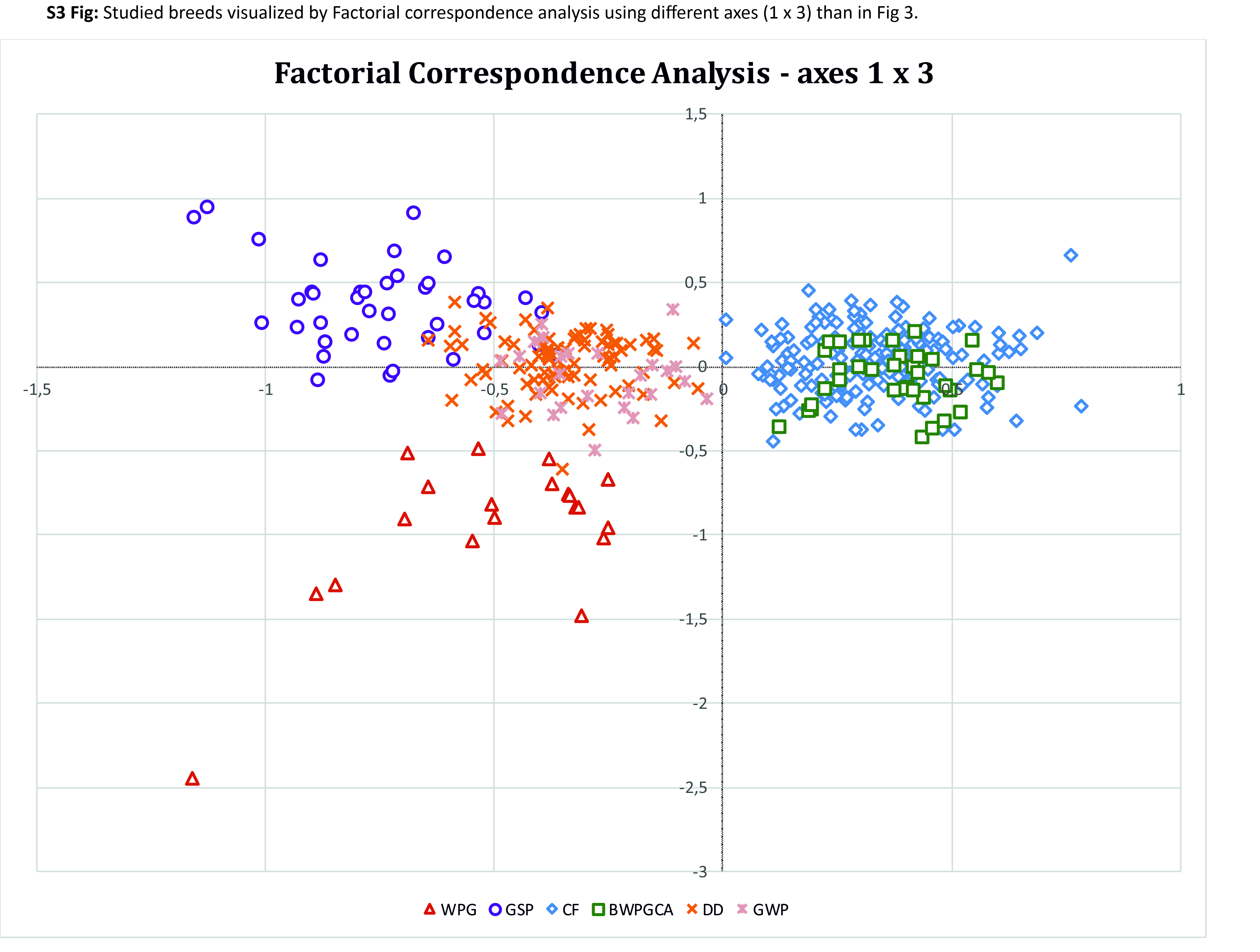

Supplement: S3 Fig — (TIF) [file pone.0221418.s003.tif]
