# Supplementary material for: Tracing genetic resurrection of pointing dog breeds: Cesky Fousek as both survivor and rescuer
Source: PLoS One. 2019 Aug 26;14(8):e0221418. doi: 10.1371/journal.pone.0221418 (PMC6709920; doi:10.1371/journal.pone.0221418)
Supplement: S2 Table — (PDF) [file pone.0221418.s005.pdf]

S2 Table. Information about hunting breeds gathered from different scientific papers.

Breed	n	H_E	Ho	Na	F_{IS}	Ar	Author
German Wirehaired Pointer	50	0.71	0.69	6.94	nd	nd	(DeNise et al., 2004)
	11	0.73	0.72	5.10	nd	nd	(Pedersen et al., 2013)
German Shorthaired Pointer	148	0.69	0.68	7.35	nd	nd	(DeNise et al., 2004)
	30	0.70	0.69	nd	0.018	5.4	(Leroy et al., 2009)
	36	0.72	0.71	6.2	nd	nd	(Pedersen et al., 2013)
Wirehaired Pointing Griffon	33	0.57	0.62	4.94	nd	nd	(DeNise et al., 2004)
	27	0.69	0.71	nd	-0.027	5.0	(Leroy et al., 2009)
Pointer	50	0.68	nd	nd	nd	nd	(Parra et al., 2008)
	20	0.62	0.57	nd	0.080	4.8	(Leroy et al., 2009)
	78	0.64	0.60	5.82	nd	nd	(DeNise et al., 2004)
English Setter	66	0.62	nd	nd	nd	nd	(Parra et al., 2008)
	20	0.66	0.64	nd	0.027	5.1	(Leroy et al., 2009)
	125	0.48	0.45	5.29	nd	nd	(DeNise et al., 2004)
English Setter (show)	36	0.46	0.43	3.7	nd	nd	(Pedersen et al., 2013)
English Setter (field)	13	0.66	0.69	4.7	nd	nd	(Pedersen et al., 2013)
Irish Red White Setter	30	0.7	0.65	nd	0.069	5.3	(Leroy et al., 2009)
Irish Setter	132	0.65	0.63	6.12	nd	nd	(DeNise et al., 2004)
	17	0.63	0.62	4.8	nd	nd	(Pedersen et al., 2013)
Gordon Setter	149	0.57	0.55	5.18	nd	nd	(DeNise et al., 2004)
Vizsla	116	0.68	0.67	6.00	nd	nd	(DeNise et al., 2004)
Weimaraner	24	0.64	0.66	nd	-0.033	4.3	(Leroy et al., 2009)
	36	0.61	nd	nd	nd	nd	(Irion et al., 2003)
	50	0.72	0.65	6.90	0.059	nd	(Streitberger et al., 2012)
	88	0.54	0.54	5.24	nd	nd	(DeNise et al., 2004)
Labrador Retriever	16	0.56	0.54	nd	0.042	3.3	(Björnerfeldt et al., 2008)
	22	0.60	0.58	nd	0.021	4.4	(Leroy et al., 2009)
	50	0.48	0.35	3.30	nd	nd	(Zajc et al., 1997)
	52	0.66	0.65	5.76	nd	nd	(DeNise et al., 2004)
	44	0.64	nd	nd	nd	nd	(Irion et al., 2003)
Bracco Italiano	72	0.64	0.59	6.43	0.060	nd	(Ciampolini et al., 2011)
Brittany Spaniel	16	0.66	nd	nd	nd	nd	(Parra et al., 2008)
	44	0.66	nd	nd	nd	nd	(Irion et al., 2003)
	64	0.64	0.64	5.65	nd	nd	(DeNise et al., 2004)
	30	0.68	0.71	nd	-0.032	5.0	(Leroy et al., 2009)
	72	0.65	0.63	6.50	nd	nd	(Pedersen et al., 2013)
Dachshunds	632	0.74	0.67	7.60	0.047	nd	(Přibáňová et al.,

							2009)
	130	0.72	0.64	7.88	nd	nd	(DeNise et al., 2004)
Hanover Hound	92	0.66	0.67	6.44	-0.012	nd	(Lüpke and Distl, 2005)

References:

Björnerfeldt, S., Hailer, F., Nord, M., Vilà, C., 2008. Assortative mating and fragmentation within dog breeds. BMC Evol. Biol. 8, 28. https://doi.org/10.1186/1471-2148-8-28

Ciampolini, R., Cecchi, F., Bramante, A., Casetti, F., Presciuttini, S., 2011. Genetic variability of the Bracco Italiano dog breed based on microsatellite polimorphism. Ital. J. Anim. Sci. 10, e59. https://doi.org/10.4081/ijas.2011.e59

DeNise, S., Johnston, E., Halverson, J., Marshall, K., Rosenfeld, D., McKenna, S., Sharp, T., Edwards, J., 2004. Power of exclusion for parentage verification and probability of match for identity in American kennel club breeds using 17 canine microsatellite markers. Anim. Genet. 35, 14–17. https://doi.org/10.1046/j.1365-2052.2003.01074.x

Irion, D.N., Schaffer, A.L., Famula, T.R., Eggleston, M.L., Hughes, S.S., Pedersen, N.C., 2003. Analysis of Genetic Variation in 28 Dog Breed Populations With 100 Microsatellite Markers. J. Hered. 94, 81–87. https://doi.org/10.1093/jhered/esg004

Leroy, G., Verrier, E., Meriaux, J.C., Rognon, X., 2009. Genetic diversity of dog breeds: within-breed diversity comparing genealogical and molecular data. Anim. Genet. 40, 323–332. https://doi.org/10.1111/j.1365-2052.2008.01842.x

Lüpke, L., Distl, O., 2005. Microsatellite marker analysis of the genetic variability in Hanoverian Hounds. J. Anim. Breed. Genet. 122, 131–139. https://doi.org/10.1111/j.1439-0388.2005.00501.x

Parra, D., Méndez, S., Cañón, J., Dunner, S., 2008. Genetic differentiation in pointing dog breeds inferred from microsatellites and mitochondrial DNA sequence. Anim. Genet. 39, 1–7. https://doi.org/10.1111/j.1365-2052.2007.01658.x

Pedersen, N., Liu, H., Theilen, G., Sacks, B., 2013. The effects of dog breed development on genetic diversity and the relative influences of performance and conformation breeding. J. Anim. Breed. Genet. 130, 236–248. https://doi.org/10.1111/jbg.12017

Přibáňová, M., Horák, P., Schröffelová, D., Urban, T., Bechyňová, R., Musilová, L., 2009. Analysis of genetic variability in the Czech Dachshund population using microsatellite markers. J. Anim. Breed. Genet. 126, 311–318. https://doi.org/10.1111/j.1439-0388.2008.00772.x

Streitberger, K., Schweizer, M., Kropatsch, R., Dekomien, G., Distl, O., Fischer, M.S., Epplen, J.T., Hertwig, S.T., 2012. Rapid genetic diversification within dog breeds as evidenced by a case study on Schnauzers. Anim. Genet. 43, 577–586. https://doi.org/10.1111/j.1365-2052.2011.02300.x

Zajc, I., Mellersh, C.S., Sampson, J., 1997. Variability of canine microsatellites within and between different dog breeds. Mamm. Genome 8, 182–185. https://doi.org/10.1007/s003359900386
